# A comparative immunofluorescence analysis of three clinical-stage antibodies in head and neck cancer

**DOI:** 10.1186/1758-3284-3-25

**Published:** 2011-05-08

**Authors:** Kathrin Schwager, Alessandra Villa, Christoph Rösli, Dario Neri, Maria Rösli-Khabas, Gerhard Moser

**Affiliations:** 1 Philochem AG, c/o ETH Zurich, Institute of Pharmaceutical Sciences, Wolfgang-Pauli-Str. 10 HCI E520, CH-8093 Zurich, Switzerland; 2 Institute of Pharmaceutical Sciences, ETH Zurich, Wolfgang-Pauli-Strasse 10, CH-8093 Zurich, Switzerland; 3 Department of Otorhinolaryngology, Paracelsus Medical University, Federal Hospital Salzburg, Muellner-Hauptstrasse 48, A-5020 Salzburg, Austria

## Abstract

**Background:**

The antibody-based targeted delivery of bioactive molecules to tumour vasculature is an attractive avenue to concentrate therapeutic agents at cancer sites, while sparing normal organs. L19, F8 and F16 are three fully human monoclonal antibodies, specific to splice isoforms of fibronectin and tenascin-C, which bind to sites of active tissue remodeling and which are currently in Phase I and II clinical trials as radio-immunoconjugates and immunocytokines in patients with cancer and arthritis.

In this article, we report the first comparative analysis of expression patterns for the extra domains EDB and EDA of fibronectin and A1 of tenascin-C in both primary and metastatic head and neck cancer lesions.

**Methods:**

We performed a comparative immunofluorescence analysis with the L19, F8 and F16 antibodies in 40 freshly frozen human head and neck cancer specimens.

**Results:**

On average, F8 and F16 exhibited similar staining intensities, which were typically stronger than L19. Interestingly, some specimens exhibited striking differences in staining by the three antibodies.

**Conclusions:**

These results suggests that an individualized treatment procedure (e.g., choice of L19, F8 or F16 based on immuno-PET or immunofluorescence procedure) may represent the most logical avenue for offering the best possible antibody to any given patient.

## Background

Antibody-based anti-cancer pharmacodelivery strategies critically rely on the availability of good-quality tumour markers, allowing a clear differentiation between diseased tissue and healthy organs. Markers of the tumour neovasculature are particularly attractive, because of the dependence of tumours on new blood vessels to sustain growth and invasion, and because of the accessibility of these structures from the bloodstream. Furthermore, angiogenesis is a common feature of virtually all malignancies [1,2].

The alternatively spliced extra domain A (EDA) and B (EDB) of fibronectin and A1 domain of tenascin-C represent three of the best-characterized markers of angiogenesis and have been reported to be expressed around the neo-vasculature and in the stroma of virtually all types of aggressive solid tumours. Three human monoclonal antibodies specific to these targets have been developed by our groups and moved to clinical trials: L19 (specific to EDB) [3], F8 (specific to EDA) [4] and F16 (specific to the A1 domain of tenascin-C) [5]. Several antibody derivatives, based on the modification of L19, F8 or F16 with cytokines or iodine radionuclides, are currently investigated in Phase I and Phase II clinical trials in patients with cancer and with rheumatoid arthritis [6,7]. These biopharmaceuticals are called L19-^124^I, L19-^131^I, L19-IL2, L19-TNF, F8-IL10, F16-^124^I, F16-^131^I, F16-IL2, indicating the modular nature of these derivatives, in which the antibody moiety is used to deliver a payload at the site of disease.

The staining patterns of the L19 and F16 antibody in head and neck tumours [5,8] have previously been published by our group in separate articles, but the relative staining intensities of the two antibodies have so far not been compared. Furthermore, a radioiodinated version of the L19 antibody has been tested in scFv format in a small immunoscintigraphic clinical study in patients with head and neck squamous cell carcinomas (SCC), observing tumour localization in 4 of 5 patients [9]. More recently, radioiodinated derivatives of both L19 and F16 antibodies in SIP format [5,10] have been studied in Phase II clinical trials in over 100 patients with cancer, including head and neck cancer patients [6][unpublished results].

In this article, we report the first comparative analysis of expression patterns for the extra domains EDB and EDA of fibronectin and A1 of tenascin-C in both primary and metastatic head and neck cancer lesions.

## Methods

### Specimens

A total of 40 freshly frozen OCT embedded tissue samples were obtained from the University Hospital of Salzburg (Austria), Department of ear-nose-throat diseases. This study was examined by the ethical committee of the province Salzburg and patient consents for the tissue collection were obtained prior to surgery. The specimens were collected during major head and neck tumour operations (table 1). The tissues were stored at -80°C. Sections of 10 μm were cut.

**Table 1:** Description of samples used for the immunofluorescence analysis

**Sample No**.	Primary tumor/ LN metastasis^1^	Tissue origin	Histological result^2^
1	tumour	parotid gland	PA
2	metastasis	lymph node	SCC
3	tumour	hypopharynx	SCC
4	tumour	hypopharynx	SCC
5	tumour	uvula	SCC
6	tumour	skin (left auricle)	BCC
7	metastasis	lymph node	SCC
8	tumour	hypopharynx	SCC
9	metastasis (from 8)	lymph node	SCC
10	metastasis	lymph node	SCC
11	tumour	skin (nose)	SCC
12	tumour	hypopharynx	SCC
13	tumour	oral cavity (tongue)	SCC
14	metastasis (from 13)	lymph node	SCC
15	metastasis (from 8)	lymph node	SCC
16	tumour	parotid gland	PA
17	tumour	oropharynx	SCC
18	tumour	oropharynx	SCC
19	tumour	parotid gland	PA
20	tumour	parotid gland	PA
21	tumour	hypopharynx	SCC
22	tumour	larynx (vocal cord)	SCC
23	tumour	larynx	SCC
24	tumour	larynx	SCC
25	metastasis	lymph node	CS
26	tumour	oropharynx (tonsil)	SCC
27	tumour	larynx	SCC
28	tumour	oropharynx	SCC
29	tumour	supraglottis	SCC
30	tumour	supraglottis	SCC
31	tumour	supraglottis	SCC^3^
32	tumour	oral cavity (lower jaw - alveolar crest)	SCC
33	tumour	larynx (glottis - posterior commissure)	SCC
34	tumour	oral cavity (palatal arch)	SCC
35	tumour	nasopharynx	SCC
36	metastasis	lymph node	PDTC
37	metastasis	lymph node	SCC
38	tumour	oropharynx (vallecula epiglottica)	SCC
39	tumour	larynx (epiglottis)	SCC
40	tumour	larynx (epiglottis)	SCC

^1 ^Indication of primary tumour sample (LN: cervical lymph node). ^2 ^Abbreviations: SCC: squamous cell carcinoma; PA: pleomorphic adenoma (benign); CS: carcinosarcoma; PDTC: poorly differentiated thyroid carcinoma; BCC: basal cell carcinoma. ^3 ^Status post radiotherapy.

### Immunofluorescence staining

The L19 antibody, specific to the extra domain B (EDB) of fibronectin, the F8 antibody, specific to the extra domain A (EDA) of fibronectin, and the F16 antibody, specific to the extra domain A1 of tenascin-C, have been described before [3-5]. All antibodies were isolated from ETH2 antibody libraries, affinity matured, had dissociation constants in the low nanomolar range and exhibited kinetic dissociation constants *k*_off _towards the respective antigens <10^-2^s^-1 ^in real-time interaction analysis experiments on a BIAcore 3000 instrument (GE Healthcare, Zurich, Switzerland). As a negative control the antibody KSF was used. KSF is specific to hen egg lysozyme and does not show any specificity towards human antigens. All antibodies were biotinylated and carried a comparable number of biotin molecules.

For immunofluorescence, a double staining for EDA, EDB resp. TnC-A1 and von Willebrand factor was performed. The following primary antibodies were used: biotinylated F8, L19, F16 and KSF (negative control antibody) in small immunoprotein format (SIP) (2 μg/ml for all four antibodies) and polyclonal rabbit anti-human von Willebrand factor (Dako, Glostrup, Denmark). For detection of the biotinylated SIPs Streptavidin Alexa Fluor 488 (Invitrogen, Basel, Switzerland) was used. As secondary detection antibody for the anti-von Willebrand factor antibody Alexa Fluor 488 goat anti-rabbit (Invitrogen, Basel, Switzerland) antibody was used.

Cryosections were fixed in chilled aceton, rehydrated in PBS (100 mM NaCl, 30 mM Na_2_HPO_4_, 20 mM NaH_2_PO_4_, pH 7.4) and blocked with 20% goat serum in PBS. Primary antibodies were added in a final concentration of 2 μg/ml in 3% bovine serum albumin (BSA)/PBS solution and incubated for 1 h. After washing in PBS secondary antibodies were added in 3% bovine serum albumin (BSA)/PBS solution and incubated for 1 h. Sections were washed in PBS and mounted with fluorescent mounting medium (Dako, Glostrup, Denmark) and analyzed with a confocal laser scanning microscope (LSM 510 META, Zeiss, Oberkochen, Germany). Microscopic exposure times were identical for all antibodies. Images were further processed using the ImageJ software http://rsb.info.nih.gov/ij.

### Semi-quantitative evaluation of results

Staining intensities of the extra domains A and B of fibronectin and the A1 domain of tenascin C were independently assessed by experienced investigators. Staining was scored using 7 scores: 0, 0.5, 1, 1.5, 2, 2.5 and 3, according to a training table, representing a selection of the corresponding staining intensities as reference. Data are expressed as the mean ± standard error of the mean.

## Results and Discussion

**Figure 1:**
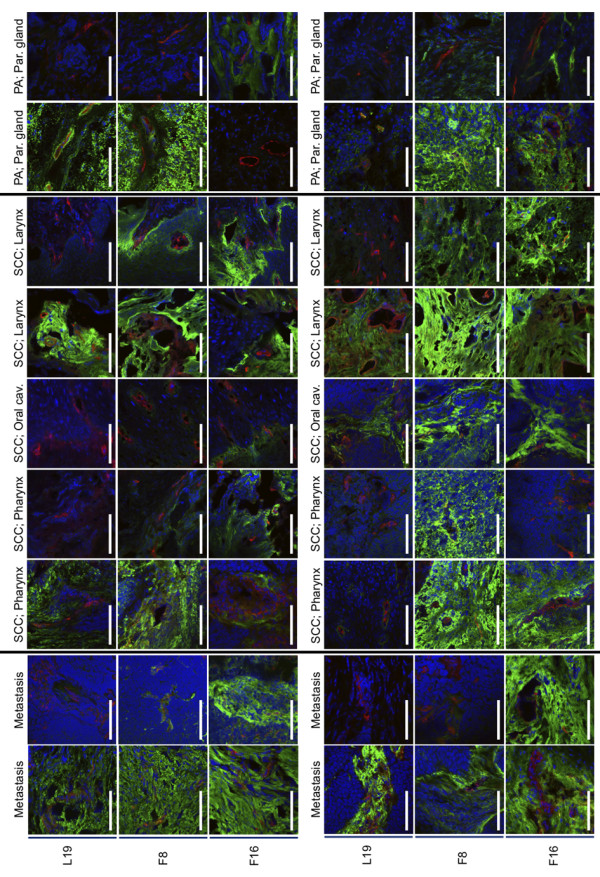
**Immunofluorescence staining of head and neck cancer samples**. The expression patterns and intensities of the splice isoforms extra domain A (EDA) and extradomain B (EDB) of fibronectin and the A1 domain of tenascin C were detected with the antibodies F8, L19 and F16, respectively (shown in green). A co-staining with an anti-von Willebrand factor antibody that stains blood vessels (red) and Dapi (blue) was performed. Abbreviations: SCC: squamous cell carcinoma; PA: pleomorphic adenoma.

The staining intensities of biotinylated derivatives of the L19, F8 and F16 antibodies in 40 frozen specimens of head and neck cancer were studied by three-colour immunofluorescence microscopy. The tissue series included tumours of the oral cavity, the pharynx and the larynx [table 1]. Primary benign lesions, malignant tumours as well as metastatic cancer specimens were analyzed. The three antibodies L19, F8 and F16 had comparable high affinities towards their cognate antigen and were used in identical concentrations. The KSF antibody, specific to hen egg lysozyme and with no known reactivity towards human antigens [11], was used as negative control (data not shown). Figure 1 shows representative immunofluorescence findings with the three antibodies (green) in different tumour sections. Cell nuclei were stained with DAPI (blue), while blood vessels were coloured in red using an anti-von Willebrand antibody. In most specimens, all three antibodies exhibited a diffuse stromal staining, similar to previously described patterns of fibronectin and tenascin-C expression [12,13]. Interestingly, some specimens exhibited striking differences in staining by the three antibodies [Figure 1]. This observation suggests that, while at least one of the three antibodies is generally capable of intensely staining a given tumour, an individualized treatment procedure (e.g., choice of L19, F8 or F16 based on immuno-PET or immunofluorescence procedure) may represent the most logical avenue for offering the best possible antibody to any given patient.

**Figure 2:**
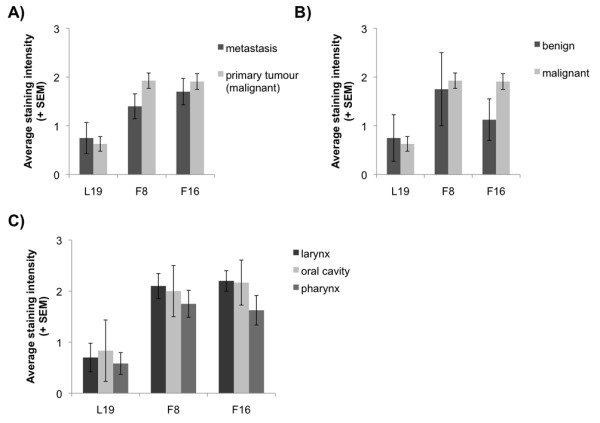
**Analysis of staining intensities**. Comparison of staining intensities obtained with the antibodies F8, L19 and F16 in A) primary tumours and metastasis tumour samples and in B) primary benign and primary maligant tumour specimens. C) Average staining intensities of tumours of the oral cavity, the pharynx or the larynx. Standard Errors of the Mean of scoring values are displayed.

Figure 2 presents a semi-quantitative analysis of staining intensities of L19, F8 and F16, ranging from 0 (absent), to 3 (+++). Independent investigators assessed all pictures, assigning a scoring intensity to each specimen. The average of these values and the corresponding standard error were displayed in the figure. Primary tumours and metastatic tumour lesions as well as primary benign and malign tumours were analyzed, as well as tumour samples from different anatomical locations (oral cavity, larynx and pharynx). On average, F8 and F16 exhibited similar staining intensities, which were typically stronger than L19. As a consequence, armed antibodies based on F8 or F16 may represent the best choice for the treatment of head and neck cancer, whereas L19 may be more appropriate for other cancer types which strongly express the EDB domain of fibronectin (e.g., kidney cancer; [7]). However, it cannot be excluded that the weaker staining observed for EDB may be a consequence of lower stability of this antigen, which has been reported in case of tissue section storage at -80°C for longer than 20 days [14]. In a previous study, a radioiodinated version of the L19 antibody has been tested in scFv format in a small immunoscintigraphic clinical study in patients with head and neck squamous cell carcinomas. Successful targeting of the primary tumour could be achieved in 4 out of 5 patients [9]. Further imaging studies are needed to reveal whether targeting of F16 and F8 is superior to L19 in head and neck cancer patients. Indeed, two Phase II clinical trials with SIP(L19) [6] and SIP(F16) labelled with ^124^I [15] are currently being performed.

## Conclusions

The majority of head and neck cancer types exhibited a diffuse pattern of expression for splice isoforms of fibronectin and of tenascin-C. The clinical stage antibodies F8 and F16 stained tumours more intensely compared to L19 and may thus represent the most suitable candidates to be used as vehicles for pharmacodelivery applications.

## Competing interests

DN is the founder and shareholder of Philogen S.p.A. (Siena, Italy), the company that owns the F8, L19 and F16 antibodies.

## Authors' contributions

KS performed the immunofluroescence stainings, evaluated the results and assisted in preparing the manuscript. DN proposed, designed, and supervised the project, and wrote and revised the manuscript. CR performed the microscopical analysis, evaluated the results and revised the manuscript. GM designed the project, performed the surgery together with MRK, and wrote and revised the manuscript. All authors read and approved the final manuscript.
